# Decidual stromal cell therapy for generalized lymphadenopathy as a special clinical manifestation of COVID‐19 infection: A case report

**DOI:** 10.1002/ccr3.5851

**Published:** 2022-05-16

**Authors:** Ali Pirsalehi, Masoud Soleimani, Abbas Hajifathali, Behnam Sadeghi, Behrouz Farhadihosseinabadi, Sedigheh Sadat Akhlaghi, Elham Roshandel

**Affiliations:** ^1^ Department of Internal Medicine School of Medicine Shahid Beheshti University of Medical Sciences Tehran Iran; ^2^ Hematopoietic Stem Cell Research Center Shahid Beheshti University of Medical Sciences Tehran Iran; ^3^ Translational Cell Therapy Research (TCR) Department of Clinical Science, Intervention and Technology CLINTEC Karolinska Instituted Huddinge Sweden

**Keywords:** COVID‐19, COVID‐19 cell therapy, decidual stromal cell, generalized lymphadenopathy

## Abstract

We are going through the greatest global health crisis of the last decades, the coronavirus disease 2019 (COVID‐19) pandemic. It may cause morbidity and mortality in some cases, and there is no therapeutic approach with reproducible and favorable outcomes. As clinical manifestations differ from patient to patient, any report regarding clinical symptoms has been beneficial for early detection and treatment. Due to the immunomodulatory effect of mesenchymal stem cells (MSCs), MSCs‐based therapy has been approved to be one of the therapeutic strategies for COVID‐19 management. For the first time in the literature, we reported generalized lymphadenopathy with fever and no sign of respiratory distress in a 16‐year‐old patient with confirmed COVID‐19 infection as the main clinical signs. We also introduce decidual stromal cells as a potential immunomodulatory treatment for COVID‐19–infected patients.

## Graphical abstract

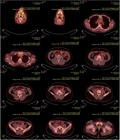

## 1 INTRODUCTION

Based on Johns Hopkins University statistics, from December 2019 to the end of March 2022, over 490 million confirmed cases of coronavirus disease 2019 (COVID‐19) have been reported all over the world, leading to more than 6 million deaths, with a case fatality rate of 1.25%.^1^ It is caused by severe acute respiratory syndrome coronavirus 2 (SARS‐CoV‐2), and the clinical manifestations mainly include fever, dry cough, fatigue, and chest pain.^2^ Human‐to‐human nosocomial transmission is the most common way of COVID‐19 spread, which is transmitted from infected persons via aerosol, airborne, or even infected surfaces.^3^ Exhalation, sneezing, and coughing generate aerosol, and inhaling the virus‐bearing aerosols can directly infect the respiratory tract of the others in the patients’ proximity.^4^ Due to improved transportation networks and globalization, human mobility is the main cause of the widespread transmission of COVID‐19, resulting in its pandemic. In the absence of widely distributed vaccines, mobility and gathering restrictions were the primary strategy to diminish the COVID‐19 transmission.^5^

Treatment strategies for COVID‐19 can be classified into three categories: (i) antiviral drugs, including Lopinavir/ritonavir, Darunavir/cobicistat, Remdesivir, Favipiravir, and Umifenovir; (ii) immunomodulatory drugs, like Chloroquine/hydroxychloroquine, Corticosteroids, Tocilizumab, Sarilumab, Anakinra, Emapalumab, Baricitinib, Mavrilimumab, Canakinumab, Colchicine, and interferons; and (iii) other drugs, such as neutralizing antibodies (intravenous immunoglobulins, hyperimmune plasma), low‐molecular‐weight heparin and unfractionated heparin, antibiotics (Azithromycin, Doxycycline), angiotensin receptor blockers and Angiotensin convertase enzyme inhibitors, and TMPRSS2 is a serine protease. The administration timing of these therapeutic agents is critical for their efficiency. For instance, although immunomodulatory drugs induce more favorable outcomes during the cytokine storm phase, antiviral agents should be ordered shortly after symptoms onset.^6^, ^7^ Mesenchymal stromal cells (MSCs) are well‐known immunomodulatory cells with promising results in controlling hyperinflammatory conditions.^8^ Decidual stromal cells (DSCs) are MSCs derived from the placenta and fetal membrane.^9^ Compared to the bone marrow‐derived MSCs, DSCs have much stronger anti‐inflammatory capacities that candidate them for cell therapy of inflammatory diseases.^9^ Their safety and promising results in controlling inflammatory disorders, such as graft‐versus‐host disease, neural inflammation, and acute respiratory distress syndrome (ARDS)^9^, ^10^, ^11^, ^12^ have led us to apply them in the case of COVID‐19–related hyperinflammation.

The clinical observations among COVID‐19–infected patients may be surprisingly different, complicating the treatment process. Hence, numerous global reports about the diversity of COVID‐19 clinical symptoms can be beneficial for early detection and on‐time treatment. Here, we report a COVID‐19–positive patient admitted to Taleghani hospital, Shahid Beheshti University of Medical Sciences, Tehran, Iran, with unusual generalized lymphadenopathy and no respiratory complications. The case is successfully treated with two intravenous doses of DSCs.

## 2 CASE PRESENTATION

A 16‐year‐old boy who had suffered from fever (38.5 °C) for the past 24 h was admitted to the Taleghani Hospital, Tehran, Iran, on May 12, 2020. The patient showed no symptoms other than fever and generalized lymphadenopathy at the admission time. The CBC result showed a reduced WBC, Hb level, and MCV and MCH indexes; conversely, neutrophil count exhibited an abnormal increase (Table 1). However, the arterial blood gases were normal, and no sign of respiratory distress was observed at the admission time. Some inflammatory markers such as IL‐6, CRP, ESR, CPK, and LDH, coagulation factors like troponin, D‐dimer, and fibrinogen, and some biochemical parameters including ferritin, AST, ALT, and ALP were evaluated at the admission day (Table 2). IL‐6, CRP, ESR, CPK, LDH, and D‐dimer parameters were higher than the reference range at the admission day, while troponin, fibrinogen, ferritin, AST, ALT, and ALP parameters were in the range.

**TABLE 1:** The results of CBC and oxygen saturation before DSC administration and after the first and second injection

Parameters and their reference range	Before DSC administration	After first DSC administration	After second DSC administration
Cell blood count			
RBC (3.8–5.1 ×10^6^ µl)	5.55	5.28	5.66
WBC (4.0–10.5× 10^3^ µl)	3.1	2.9	5.1
Lymphocyte (20–40%)	30	35	27
Neutrophil (50–70%)	67	62	70
Monocyte (3–12%)	3	2	3
Eosinophil (0.5–5%)	0	1	0
Basophil (0–1%)	0	0	0
PLT (145–449 × 10^3^ /µl)	157	189	244
RBC indexes			
HGB (14–18 g/dl)	13.7	13.2	12.7
HCT (42–52%)	42.1	39.3	38.1
MCV (80–96 fl)	75.8	74.3	67.3
MCH (27–32 pg)	25.8	25.5	23.3
MCHC (33–36 g/dl)	33.9	34.4	34.6
Oxygen saturation (95–98%)	91	90	93

**TABLE 2:** The results of some inflammatory markers, coagulation assays, and biochemical tests before DSC administration and after the first and second injection

Parameters and their reference range	Before DSC administration	After first DSC administration	After second DSC administration
IL‐6 (5–15 pg/ml)	111	32	11
CRP (<6 mg/L)	47	29	18
ESR (1–10 mm/h)	28	16	7
CPK (60–400 IU/L)	428	99	58
LDH (140–280 IU/L)	377	325	273
Troponin (0.04 ng/ml)	Negative	Negative	Negative
D‐dimer (<0.5 μ/ml)	+4	+1	Negative
Fibrinogen (200–400 mg/dl)	372	245	202
Ferritin (12–300 ng/ml)	73	69	52
AST (10–40 IU/L)	37	28	16
ALT (29–33 IU/L)	19	16	13
ALP (20–140 IU/L)	488	357	254

Initial medical examinations were performed on May 14, 2020, to assess lymphadenopathy by sonography of the neck, bilateral inguinal, abdomen, and pelvis. The results showed a few reactive lymph nodes at the jugular chain and bilateral submandibular; the most prominent lymph nodes with short‐axis diameters (SAD) of 11 and 12 mm were found in the right and left submandibular areas, respectively. Besides, several reactive lymph nodes were observed at the axillary and inguinal regions with SAD of 10 and 12.5 mm, respectively.

**FIGURE 1:**
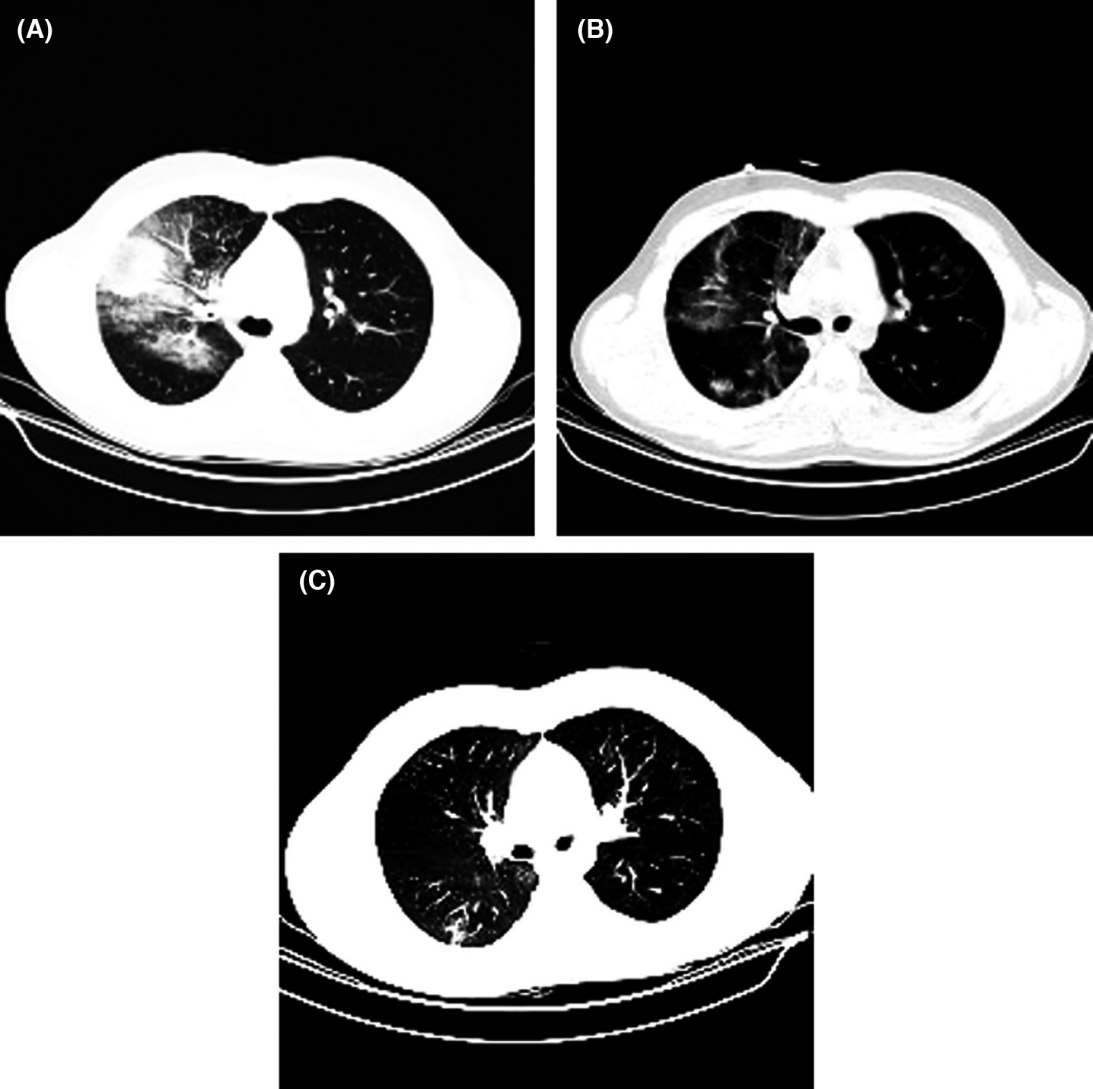
CT scan images (A) The CT result on admission day (B) The CT result 24 h after the first injection of decidual stromal cell administration (C) The CT result 24 h following the second injection of decidual stromal cells

On May 14, 2020, the spiral chest CT scan showed the bilateral multifocal ground‐glass opacities with remarkable infiltrations in the lungs that strongly suggested COVID‐19 infection as the main diagnosis. Furthermore, a mediastinal CT scan indicated hepatosplenomegaly, and bilateral axillary and bilateral lymphadenopathy with a maximum SAD of 12 and 15 mm, respectively (Figure 1). However, COVID‐19 real‐time PCR tests on nasopharyngeal and oropharyngeal samples were negative. The presence or absence of viral infection must be confirmed regarding the patient’s symptoms and lymphadenopathy. Eventually, SARS‐CoV‐2 RNA was detected in the bronchoalveolar lavage sample using real‐time PCR, which confirmed SARS‐CoV‐2 infection.

As the generalized lymphadenopathy has not been reported as a clinical sign in COVID‐19–positive patients, we considered other bacterial and viral infections or hematopoietic malignancies, including lymphoma, as the possible explanations for this phenomenon. In this light, the patient was confirmed to be negative for the varicella zoster virus, toxoplasmosis, cytomegalovirus (CMV), hepatitis B and C virus (HBV and HCV), human immunodeficiency virus (HIV), and Epstein–Barr virus. The transbronchial needle aspiration procedure was carried out for histological evaluations to find tumor cells within the lymphoid tissues. Unexpectedly, no sign of neoplasia was observed in the histological analysis.

The COVID‐19 treatment with low‐dose corticosteroids, Lopinavir/ritonavir (Kaletra) 200/50 mg, Hydroxychloroquine 400 mg, and azithromycin 250 mg was started following the COVID‐19 positive result. However, high‐dose dexamethasone was not prescribed, considering the possibility of neoplasia. Despite the COVID‐19 treatment, the blood O_2_ saturation decreased, lymphadenopathy and fever were not ameliorated, and the patient experienced a significant weight loss and disease progression.

On May 15, 2020, the patients underwent cell therapy with decidual stromal cells (DSCs). DSCs were isolated from the fetal membranes under GMP conditions based on a previously described protocol.^9^ After providing informed consent, placenta tissues were obtained from healthy mothers who underwent elective cesarean section. The donors were screened for viral infections, including HIV, HBV, HCV, and CMV viruses. After transferring to the laboratory under sterile conditions, the fetal membranes were isolated, washed with PBS, dissected, and transferred into the 50 ml falcons containing trypsin/EDTA for digestion and DSCs isolation. Then, the isolated cells were cultured in Dulbecco's modified Eagle medium (DMEM) supplemented with 10% fetal calf serum (FSC). The fetal membrane residues were cultured in the T175 flasks containing DMEM to form the fibroblast‐like cell colonies. Eventually, the cultured cells were harvested using trypsin/EDTA when they reached 90–95% confluence, then washed with complete DMEM, and cultured in new T175 flasks containing DMEM supplemented with 10% FSC. Isolated DSCs were expanded until passage five (P5). For DSCs characterization, the expression of CD105, CD90, CD73, CD44, CD45, and CD34 surface markers was examined using flow cytometry. The DSCs’ viability and the specimens’ microbial contaminations were checked prior to injection. 100 mg hydrocortisone was injected about half an hour before each DSC administration to prevent the side effects of the cell therapy. Two doses of 1–1.2 × 10^6^ cell/kg, suspended in 0.9% NaCl injection solution, were injected into the central venous line within 5 min with 4‐day interval. The vital signs were monitored every 3 h within the first 24 h following DSC injection and every 6 h within 48 h later. The patient presented no complications, even dizziness, and the most common adverse effect of cell therapy.

**FIGURE 2:**
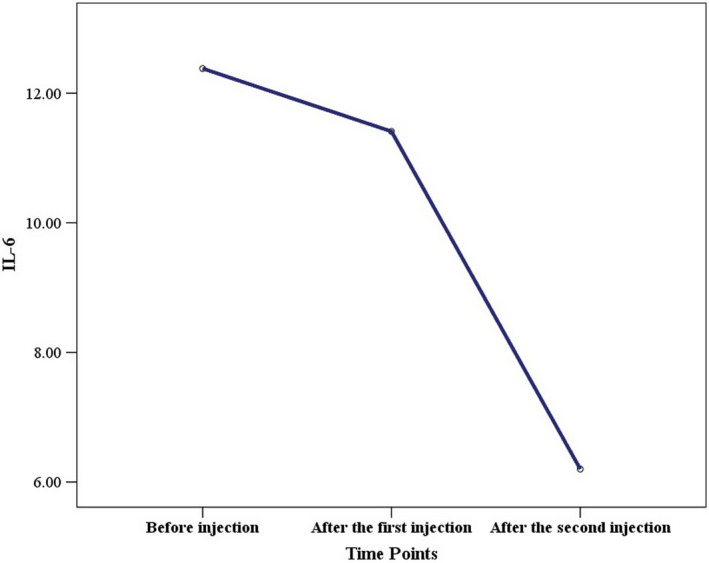
Serum IL‐6 before and after the first and second decidual stromal cell administration. The serum concentrations of IL‐6 cytokine significantly decreased after the first and second decidual stromal cell injection

Evaluation of IL‐6, as one of the main inflammatory cytokines, showed a significant decline following cell injection, where its concentration decreased from 12.38 pg/ml before cell therapy to 6.2 pg/ml after cell therapy (Figure 2). The CT scan results after the first and second doses of DSCs indicated the positive impact of this medical intervention.

**FIGURE 3:**
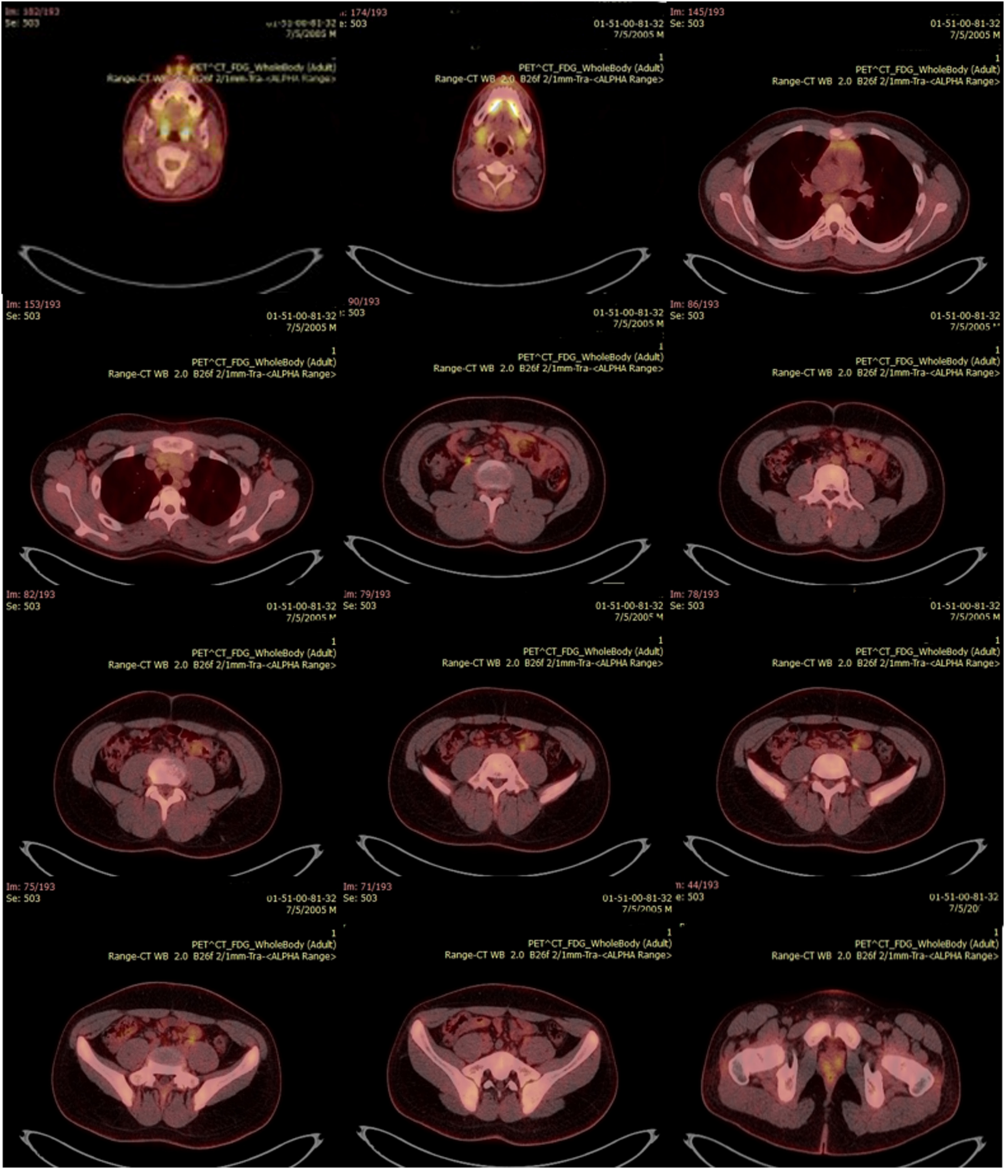
PET/CT image. The PET/CT result indicated no sign of lymph proliferative disorders in the patient

On May 20, 2020, the patient was discharged from the hospital in good general condition. The PET/CT examination was performed on July 5, 2020, to rule out any malignancies, which showed no sign of lymphoproliferative disorders in the patient (Figure 3).

The results of CBC analysis, oxygen saturation test, inflammatory markers (IL‐6, CRP, ESR, CPK, and LDH), coagulation assays (troponin, D‐dimer, and fibrinogen), and some biochemical tests (ferritin, AST, ALT, and ALP) before DSC administration and after the first and second DSC injection were presented in Tables 1 and 2. WBC count and the level of IL‐6, CRP, ESR, CPK, LDH, and D‐dimer were increased after DSC therapy.

## 3 DISCUSSION

The clinical manifestations of COVID‐19 include a wide range from mild to severe, including fever, tiredness, dry cough, and chest distress which may develop ARDS and even multiple organ failure. Lymphadenopathy is the less common symptom, along with headache, diarrhea, skin rashes, and lymphadenopathy.^2^ Although CT scan has a leading role in COVID‐19 diagnosis and management,^13^ few studies have reported the mediastinal lymphadenopathy in COVID‐19–positive patients as an atypical CT feature. Shi et al. detected lymphadenopathy in 6% of radiological analysis of 81 COVID‐19–infected patients.^14^ Valette et al. reported mediastinal lymphadenopathy in the six cases of nine COVID‐19–positive patients. They suggested a relationship between mediastinal lymphadenopathy and COVID‐19 infection severity. Accordingly, the patients with critical conditions exhibited larger mediastinal lymph nodes.^15^

Lymphadenopathy could have several etiologies, including malignancies, infection, autoimmune disorders, and iatrogenic causes.^16^ In this case, after ruling out the malignancy, COVID‐19 real‐time PCR and chest CT scan results suggested that SARS‐CoV‐2 infection was the reason for the generalized lymphadenopathy. The standard of practice in managing lymphadenopathy is to find and treat its underlying cause. We started low‐dose corticosteroids, which did not improve the patient's condition and deteriorated the patients’ symptoms. Although malignancy was ruled out, and since the generalized lymphadenopathy is not a typical symptom of COVID‐19, we preferred not to prescribe high‐dose corticosteroids due to the minimum possibility of malignancies. Therefore, the immunomodulatory approach was preferred over immunosuppressive treatments in hyperinflammatory conditions.

Regarding immunomodulatory and tissue‐repairing effects of MSCs and trapping of a considerable part of infused MSCs in lungs,^16^ they are considered promising choices for cell therapy of COVID‐19, where hyperinflammation and lung tissue degeneration are the main causes of mortality and morbidity.^17^ Numerous clinical trials are currently evaluating the efficacy of MSCs in controlling the cytokine storm and hyperinflammation of COVID‐19, and^17^, ^18^, ^19^, ^20^ their safety is approved in several studies.^18^, ^21^, ^22^ Several studies reported the effectiveness of DSCs, stromal cells with significantly stronger anti‐inflammatory properties,^23^ in controlling the hyperinflammatory disorders. First, Ringden et al. reported the safety and efficacy of DSCs and their significant privilege in alleviating inflammation.^11^, ^12^, ^23^, ^24^

This case report is the first report of using DSC infusion in a COVID‐19–infected patient with a hyperinflammatory condition. Although a one‐patient case report could not be conclusive, our results encourage using DSCs as an immunomodulatory element to attenuate hyperinflammation in COVID‐19. The patients’ lymphadenopathy was completely cured, and no abnormality was found after 1 year of monitoring. Since the patient was young, it can be considered that his improvement might be due to the clinical course of the disease rather than the DSC therapy. Since the conventional treatments for COVID‐19 did not ameliorate the fever, lymphadenopathy, and weight loss and even worsened the O_2_ saturation, it could be concluded that DSCs might, at least in part, have led to his improvement.

## 4 CONCLUSION

Despite the ineffectiveness of the conventional therapies for COVID‐19, DSCs showed a favorable effect on controlling the COVID‐19 symptoms in the presented case. However, several controlled trials are required to prove the safety and efficacy of DSC therapy in severe COVID‐19.

## AUTHOR CONTRIBUTIONS

Elham Roshandel contributed to conceptualization; Abbas Hajifathali contributed to methodology; Sedigheh Sadat Akhlaghi contributed to formal analysis and investigation; Behrouz Farhadi Hosseinabadi contributed to writing—original draft preparation; Behnam Sadeghi contributed to writing—review and editing; Masoud Soleimani contributed to funding acquisition; Ali Pirsalehi contributed to supervision. All authors revised the manuscript and approved the final paper.

## CONFLICT OF INTEREST

The authors declare no conflict of interest.

## ETHICAL APPROVAL

All medical interventions in this study have been approved by the Ethics Research Committee of Shahid Beheshti University of Medical Sciences under the ethical code number of IR.SBMU.MSP.REC.1399.118.

## CONSENT

Written informed consent was obtained from the patient to publish this report in accordance with the journal's patient consent policy.

## References

[1] WHO Coronavirus (COVID‐19) Dashboard . https://covid19.who.int

[2] Khorramdelazad H , Kazemi MH , Najafi A , Keykhaee M , Emameh RZ , Falak R . Immunopathological similarities between COVID‐19 and influenza: investigating the consequences of co‐infection. Micro Pathogen. 2021;152:104554. 10.1016/j.micpath.2020.104554 PMC7607235 33157216

[3] Khan AH , Tirth V , Fawzy M , et al. COVID‐19 transmission, vulnerability, persistence and nanotherapy: a review. Environ Chem Lett. 2021;19(4):2773‐2787. 33846683 10.1007/s10311-021-01229-4 PMC8026094

[4] Salian VS , Wright JA , Vedell PT , et al. COVID‐19 transmission, current treatment, and future therapeutic strategies. Mol Pharm. 2021;18(3):754‐771. 33464914 10.1021/acs.molpharmaceut.0c00608

[5] Zhang M , Wang S , Hu T , et al. Human mobility and COVID‐19 transmission: a systematic review and future directions. Ann GIS. 2022;22(1):1‐14.

[6] Bartoli A , Gabrielli F , Alicandro T , Nascimbeni F , Andreone P . COVID‐19 treatment options: a difficult journey between failed attempts and experimental drugs. Int Emergen Med. 2021;16(2):281‐308. 10.1007/s11739-020-02569-9 PMC7781413 33398609

[7] Venkatasubbaiah M , Reddy PD , Satyanarayana SV . Literature‐based review of the drugs used for the treatment of COVID‐19. Cur Med Res Pract. 2020;10(3):100‐109. 10.1016/j.cmrp.2020.05.013 PMC7301064 32572376

[8] Shi Y , Wang Y , Li Q , et al. Immunoregulatory mechanisms of mesenchymal stem and stromal cells in inflammatory diseases. Nat Rev Nephrol. 2018;14(8):493‐507. 29895977 10.1038/s41581-018-0023-5

[9] Aronsson‐Kurttila W , Baygan A , Moretti G , et al. Placenta‐derived decidua stromal cells for hemorrhagic cystitis after stem cell transplantation. Acta Haematol. 2018;139(2):106‐114. 29408819 10.1159/000485735

[10] Sadeghi B , Moretti G , Arnberg F , et al. Preclinical toxicity evaluation of clinical grade placenta‐derived decidua stromal cells. Front Immunol. 2019;10:2685. 31803191 10.3389/fimmu.2019.02685 PMC6877599

[11] Ringdén O , Sadeghi B , Moretti G , et al. Long‐term outcome in patients treated at home during the pancytopenic phase after allogeneic haematopoietic stem cell transplantation. Int J Hematol. 2018;107(4):478‐485. 29143281 10.1007/s12185-017-2363-5

[12] Ringden O , Solders M , Erkers T , et al. Placenta‐derived decidual stromal cells for graft‐versus‐host disease, hemorrhaging, and toxicity after allogeneic hematopoietic stem cell transplantation. Biol Blood Marrow Transplant. 2015;21(2):S149.

[13] Rubin GD , Ryerson CJ , Haramati LB , et al. The role of chest imaging in patient management during the COVID‐19 pandemic: a multinational consensus statement from the Fleischner Society. Chest. 2020;158(1):106‐116. 32275978 10.1016/j.chest.2020.04.003 PMC7138384

[14] Shi H , Han X , Jiang N , et al. Radiological findings from 81 patients with COVID‐19 pneumonia in Wuhan, China: a descriptive study. Lancet Infect Dis. 2020;20(4):425‐434. 32105637 10.1016/S1473-3099(20)30086-4 PMC7159053

[15] Valette X , du Cheyron D , Goursaud S . Mediastinal lymphadenopathy in patients with severe COVID‐19. Lancet Infect Dis. 2020;20(11):1230. 10.1016/S1473-3099(20)30310-8 PMC7173806 32330440

[16] Gaddey HL , Riegel AM . Unexplained lymphadenopathy: evaluation and differential diagnosis. Am Fam Physician. 2016;94(11):896‐903. 27929264

[17] Song N , Wakimoto H , Rossignoli F , et al. Mesenchymal stromal cell immunomodulation: in pursuit of controlling COVID‐19 related cytokine storm. Stem Cells. 2021;36(9):707 ‐722. 10.1002/stem.3354 PMC8014246 33586320

[18] Siddesh SE , Gowda DM , Jain R , et al. Placenta‐derived mesenchymal stem cells (P‐MSCs) for COVID‐19 pneumonia—a regenerative dogma. Stem Cell Investig. 2021;8:3. 10.21037/sci-2020-034 PMC7937692 33688491

[19] Golchin A , Seyedjafari E , Ardeshirylajimi A . Mesenchymal stem cell therapy for COVID‐19: present or future. Stem Cell Rev Rep. 2020;16(3):427‐433. 32281052 10.1007/s12015-020-09973-w PMC7152513

[20] Kavianpour M , Saleh M , Verdi J . The role of mesenchymal stromal cells in immune modulation of COVID‐19: focus on cytokine storm. Stem Cell Res Ther. 2020;11(1):1‐9. 32948252 10.1186/s13287-020-01849-7 PMC7499002

[21] Saleh M , Vaezi AA , Aliannejad R , et al. Cell therapy in patients with COVID‐19 using Wharton’s jelly mesenchymal stem cells: a phase 1 clinical trial. Stem Cell Res Ther. 2021;12(1):1‐3. 34271988 10.1186/s13287-021-02483-7 PMC8283394

[22] Razi S , Molavi Z , Mirmotalebisohi SA , et al. Mesenchymal stem cells in the treatment of new coronavirus pandemic: a novel promising therapeutic approach. Adv Pharm Bull. 2021;12(2):206‐216. 35620342 10.34172/apb.2022.023 PMC9106958

[23] Ringden O , Baygan A , Remberger M , et al. Placenta‐derived decidua stromal cells for treatment of severe acute graft‐versus‐host disease. Stem Cells Transl Med. 2018;7(4):325‐331. 29533533 10.1002/sctm.17-0167 PMC5866941

[24] Ringdén O . Mesenchymal stem (stromal) cells for treatment of acute respiratory distress syndrome. Lancet Respir Med. 2015;3(4):e12. 10.1016/S2213-2600(15)00047-8 25890656

